# Infrared-spectroscopic, dynamic near-field microscopy of living cells and nanoparticles in water

**DOI:** 10.1038/s41598-021-01425-w

**Published:** 2021-11-08

**Authors:** Korbinian J. Kaltenecker, Thorsten Gölz, Enrico Bau, Fritz Keilmann

**Affiliations:** 1 Fakultät für Physik, Nano Institute Munich & Center for NanoScience (CeNS), Ludwig-Maximilians-Universität, Königinstr. 10, 80539 Munich, Germany; 2 Present Address: Attocube Systems AG, Eglfinger Weg 2, 85540 Haar, Germany

**Keywords:** Scanning probe microscopy, Infrared spectroscopy, Molecular imaging, Cellular microbiology, Super-resolution microscopy

## Abstract

Infrared fingerprint spectra can reveal the chemical nature of materials down to 20-nm detail, far below the diffraction limit, when probed by scattering-type scanning near-field optical microscopy (s-SNOM). But this was impossible with living cells or aqueous processes as in corrosion, due to water-related absorption and tip contamination. Here, we demonstrate infrared s-SNOM of water-suspended objects by probing them through a 10-nm thick SiN membrane. This separator stretches freely over up to 250 µm, providing an upper, stable surface to the scanning tip, while its lower surface is in contact with the liquid and localises adhering objects. We present its proof-of-principle applicability in biology by observing simply drop-casted, living *E. coli* in nutrient medium, as well as living A549 cancer cells, as they divide, move and develop rich sub-cellular morphology and adhesion patterns, at 150 nm resolution. Their infrared spectra reveal the local abundances of water, proteins, and lipids within a depth of ca. 100 nm below the SiN membrane, as we verify by analysing well-defined, suspended polymer spheres and through model calculations. SiN-membrane based s-SNOM thus establishes a novel tool of live cell nano-imaging that returns structure, dynamics and chemical composition. This method should benefit the nanoscale analysis of any aqueous system, from physics to medicine.

## Introduction

Visible-light microscopy has through decades produced sensational motion pictures of living systems, based on Leeuwenhoek’s 1677 discovery of swimming bacteria^1^, up to viewing rotating protein machines^2^. The initial, poor *intensity* contrasts could be improved by acquiring *phase* contrasts of the transmitted light, but only the incorporation of fluorescent dye molecules into living systems delivers the awesome, state-of-the-art *colour* contrasts that discriminate sub-cellular structures such as lipid membranes, nuclei, protein clusters, or organelles down to single molecules. Fluorescence especially has furthermore enabled a tenfold improvement of the microscopic resolution far below the Abbe diffraction limit (given by *λ*/2, where *λ* is the wavelength) to around 20 nm, through exploiting nonlinear responses in dye molecules^3^. Other super-resolution microscopy techniques such as photoactivated localization microscopy (PALM)^4^ share the need for fluorescent-molecular labels, which may perturb biological dynamics and photo-bleach.

Infrared microscopy distinguishes itself from these methods in two major ways. On one hand, it suffered initially from its much larger diffraction limit (around 5 µm for *λ* ≈ 10 µm). But on the flipside, infrared spectra offer intrinsic *molecular colour* contrasts, notabene without labelling, stemming from absorption resonances which occur in any organic material. Pertaining spectra in the "fingerprint region" of wavelengths between 5 and 15 µm routinely determined by FTIR (Fourier-transform infrared) spectroscopy serve for quantitative chemical recognition in many fields of chemical analytics. This potential is also gaining increasing interest in the life sciences where, for example, advanced workflow procedures can classify embedded tumour tissue sections on the basis of minute spectral differences^5^.

**Figure 1:**
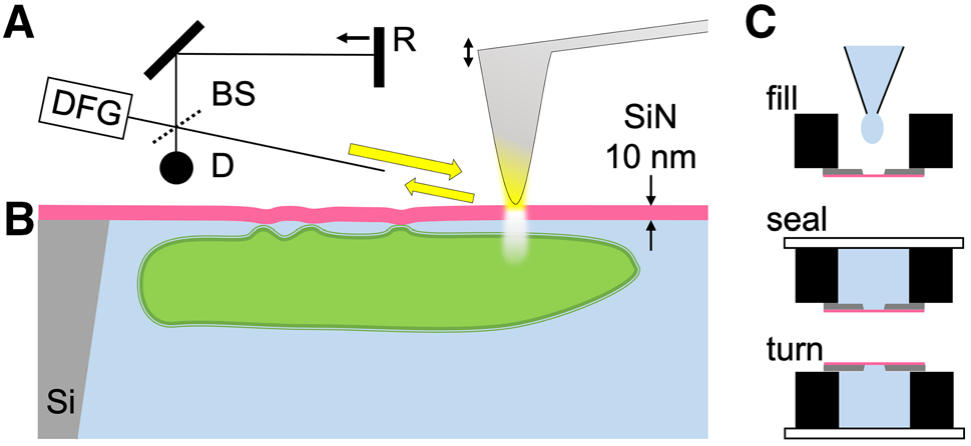
SiN-membrane-based infrared probing into liquids, (**a**) nano-FTIR spectroscopic near-field microscopy layout using light focused on a metallic AFM tip to induce an intense near-field spot which is as narrow as the tip and extends a similar distance below the tip apex (white patch)^8,44^. A sample scanned below the tip, through the near-field spot, modifies the back-scattered light which is detected at the detector (D), via a Michelson interferometer that employs a beam splitter (BS) and a movable reference mirror (R) to form highly resolved infrared s-SNOM images. When the light is broad-banded, e.g., from a DFG (difference-frequency generation) source, a nano-FTIR spectrum can be measured at any sample position, in both scattered amplitude and phase. In addition, the tapping tip (double arrow) may sense membrane surface deformations via the AFM circuitry. (**b**,**c**) Liquid-sample cell consisting of a small metal container (black) carrying a perforated Si chip (grey) that is closed by a 10-nm thin SiN membrane (pink), loaded by drop-casting a suspension and waiting for particles or cells to settle and adhere to the membrane (top), then sealed (mid) and finally turned upside down (bottom) for microscopy so that the probing tip touches the upper surface of the membrane. When particles or cells adhere to its lower surface, the membrane can become locally distorted, as illustrated, and thus can enable a mapping of the field of adhesion forces.

Fortunately, the introduction of near-field techniques in the infrared range has enabled an even hundred-to-thousandfold improvement of the microscopic resolution below the diffraction limit, to around 20 nm. This near-field concept combines (i) plasmonic focusing of infrared light by irradiating a metallic AFM tip, thus generating a hot spot whose width is as small as the curvature radius of the tip apex, with (ii) detecting tip-scattered light which becomes characteristically encoded by interacting with a sample just under the apex. Such AFM-based s-SNOM succeeded in a first proof-of-principle demonstration of chemical contrast at 50 nm resolution using distinct wavelengths near 10 µm^6^. Veritable broad-banded "nano-FTIR" fingerprint spectra can likewise be obtained by using an infrared continuum source and interferometric detection as depicted in Fig. 1a^7–10^. Commercially available s-SNOMs complemented by nano-FTIR capability routinely resolving 30 nm have given new insights in many fields, from extaterrestrial minerals^11^ to ultrathin polymers^12^ and, as an example from biophysics, when mapping dried insulin samples' secondary-structure changes along single protofibrils^13^. A pertaining infrared-vibrational nanospectroscopic study of prion proteins undergoing conformational transitions^14^ would be highly interesting but requires, as all living matter, the presence of water.

Unfortunately, wet, living biological objects could not be directly probed by s-SNOM, because they would contaminate the tip by an undefined water meniscus, apart from possibly deteriorating by evaporation. To circumvent such problems, we proposed covering wet objects by a thin membrane, and indeed demonstrated that monoatomic graphene is in principle suited for nano-FTIR spectroscopy of a liquid sample^15^, by encapsulating virus suspensions between two graphene sheets, a method known from electron microscopy^16,17^. However, the strong inter-sheet attraction seemingly squeezes water out and compresses viruses to 60% of their native thickness, or compresses bacterial cells between Si and graphene to 14%^18^, hence this method is rather not suited for studying alive cells. Recently, single graphene or oxide sheets were used to cover liquid containers for nano-FTIR spectroscopy of liquids^19,20^, and with suspended protein aggregates^21^, but not with living objects. Here we propose a reliable, robust and easy-to-use wet cell for s-SNOM and demonstrate highly repeatable imaging and high spectral quality, based on a commercially available SiN membrane also known from electron microscopy, well suited for observing the dynamics of living biological objects.

## Experimental setup

An infrared-transparent, thin SiN-membrane cover is used for culturing, nanoscopically observing and spectroscopically characterising living cells in their native environment. The sample container used in this proof-of-principle demonstration is based on a 4 mm high Al block of 15 × 20 mm footprint and 3 mm central bore (Fig. 1b,c). A commercially available 200 µm thick Si chip (grey) with 5 mm outer diameter comes with a thin SiN top layer (pink) that forms a free-standing membrane over a central bore (norcada.com). The chip is glued to the cell using 125 µm thick double-sided adhesive tape. We chose the SiN membrane over graphene for its ready-to-use availability at modest cost, its hydrophilicity after UV treatment, and its robustness even at the 250 µm size needed to accommodate large mammalian cells. This basic concept has worked already with microwaves in a study that employed a thin SiO_2_ cover for near-field mapping of *E. coli* immobilised in glycerol solution^22^. Our present setup constitutes the first practical liquid cell for s-SNOM and nano-FTIR. It is cheap and robust, easy to fill and seal permanently, and just as easily placed and aligned on the s-SNOM platform like any other sample.

## Results

### SiN-membrane-based wet cell performance

**Figure 2:**
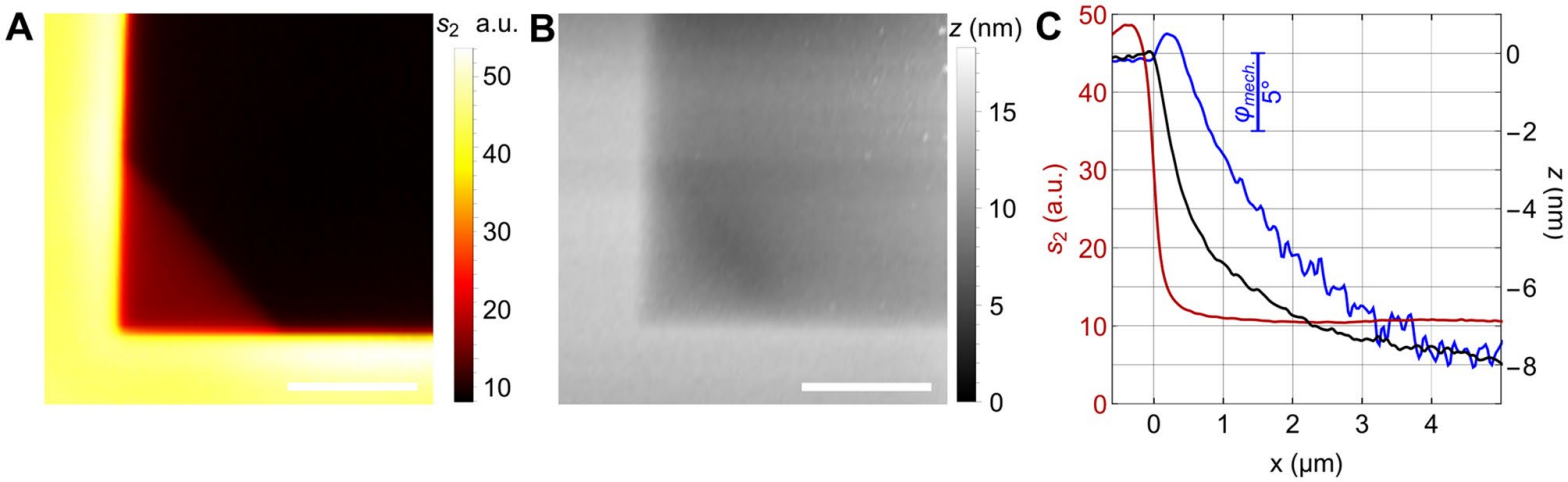
s-SNOM-observed membrane wetting, (**A**) infrared amplitude *s*_*2*_ and (**B**) topography *z* nanoimages of a free-standing 20-nm thick SiN membrane on its Si frame (scale bars 2 µm), as-received exhibits a partial wetting in the lower left corner of the 20 × 20 µm^2^ hole (Norcada NX5002Y), (**C**) after UV hydrophilisation and complete wetting, line profiles are extracted from images (not shown) of infrared amplitude *s*_*2*_ (red), topography *z* (black), and mechanical phase *φ*_*mech*_ (blue) across an edge (at *x* = 0) of the frame into the membrane.

The experiment uses a commercial near-field microscope (NeaSNOM from attocube.com) equipped with broad-band mid-infrared illumination (DFG) as sketched in Fig. 1a, and proprietary "nano-FTIR" tips (characterised to have a radius *r* ≈ 60 nm)^23^ which tap at circa 70 nm amplitude. The infrared amplitude *s*_*2*_ plotted in Fig. 2A,C for a partly water-filled SiN cell refers to the infrared signal component at the 2nd harmonic of the tapping frequency of circa 300 kHz (“Methods”), and represents an average over the band emitted by the DFG source, ranging from ca. 1100 to 1700 cm^−1^ (Supplementary Fig. S1). Mechanical images of topography *z* and mechanical phase *φ*_*mech*_ are simultaneously acquired^8^.

The AFM tapping-mode operation is observed to work stably on the membrane whether it is dry or wet on its underside (Fig. 2A). At the holes' edges, the membrane is deformed and probably dilated by van der Waals attraction forces to the side walls, as is known from graphene^24^. The topographical kink (black in Fig. 2C) at the edge appears circa 50 nm wide. This value indicates the AFM resolution, which matches the nominal tip radius. The infrared trace (red) does not show this kink, which assures that the optical channel is free of a topography-induced artefact. Its transition from a high signal on the frame to one that is about 5 × lower on the wet membrane determines an infrared 20-to-80% edge resolution of circa 150 nm. Along the water/air border on the partially wet surface, the topography exhibits a depression a few nm deep (Fig. 2B). We conclude that the membrane can flexibly respond to water's surface tension, and that this enables a welcome new channel of observing adhering living cells via adhesion maps (see below).

### Near-field spectra of water

The usable spectral range of the nano-FTIR has been set to extend from 950 to 1850 cm^−1^ where the power density of the illuminating coherent beam falls below 5% of its maximum (Supplementary Fig. S1). However, the lower frequency part of this range becomes dominated by the phonon resonance of SiN. Outside this resonance covering circa 800 to 1200 cm^−1^, the SiN-based nano-FTIR spectroscopy should perform unattenuated up to visible and down to THz frequencies.

**Figure 3:**
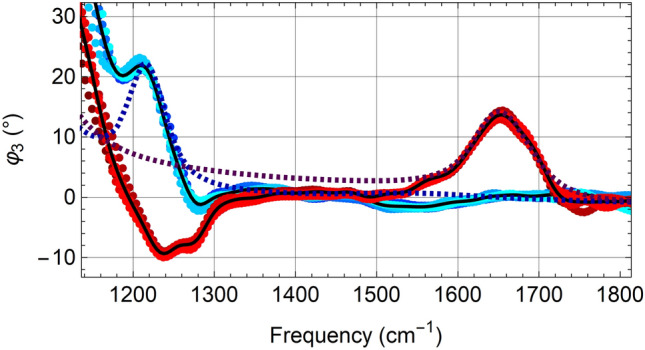
Nano-FTIR spectra of H_2_O and D_2_O compared to theoretical calculations, below a free-standing, 15-nm SiN membrane stretched over a 20 × 20 µm^2^ hole (Norcada NBPX5002YZ-HR), of infrared phase *φ*_*3*_ (round data points with black average curves) exhibiting the bending-vibrational resonances of H_2_O (red) or D_2_O (blue), at nominally 1644 and 1209 cm^−1^, respectively^61^ (see Supplementary Fig. S2 for simultaneously measured amplitude spectra, and for an H_2_O/D_2_O mixture). The theoretically predicted curves (square dots) for H_2_O and D_2_O under 15-nm SiN are calculated using the finite-dipole model of tip-confined near-field interaction extended to multi-layer objects (“Methods”), assuming a 100 nm tip radius and 70 nm tapping amplitude, plotted after multiplication by 1.78.

The influence of a 15-nm SiN cover can be best recognised in the experimental spectrum of H_2_O (red in Fig. 3) as a rising edge below 1300 cm^−1^, where H_2_O has no resonance. Interestingly, the D_2_O resonance at 1209 cm^−1^ remains visible where expected, and appears as an addition to a steep slope marking the edge of the phonon resonance of SiN. The model calculation (square dots in Fig. 3) supports the notion of additivity of spectra even though it does not agree with the steepness of the measured SiN slope; possibly the SiN dielectric function^25^ used for our calculation does not apply to the Norcada product, given that this material is non-stoichiometric, amorphous and prone to vary with production parameters.

Above 1300 cm^−1^ the *φ*_*3*_ phase spectrum of D_2_O is flat with spurious features < 1° rms which assures that SiN-based nano-FTIR in this spectral region produces reliable infrared spectra with no need of correction. The excellent agreement between experiment and theory for the 1644 cm^−1^ resonance profile of H_2_O testifies to the spectroscopic accuracy attained. The width of this resonance is 75 cm^−1^ (FWHM), well-resolved with our instrumental resolution of circa 17 cm^−1^. Its peak height is 14°, in close agreement with a previous s-SNOM measurement of H_2_O through monolayer graphene where 8° in *φ*_*2*_ was obtained, using a NeaSNOM with incoherent synchrotron illumination^19^.

### Depth range of nano-FTIR sensing

**Figure 4:**
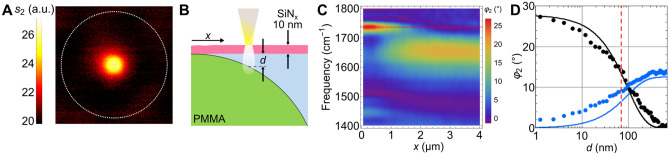
Calibrating the depth sensitivity of s-SNOM imaging and nano-FTIR spectroscopy by probing a 10 µm diameter PMMA sphere (PMMA-F-10.0 from micro-particles.de) adhering below a 10 nm SiN membrane (Norcada NX5025Z) in water, (**A**) infrared amplitude *s*_*2*_ image with marked outline of the sphere, (**B**) sketch of taking a line scan of spectra in 48 nm sequence along a radius *x* of the projected sphere, resulting in (**C**) nano-FTIR phase *φ*_*2*_ spectra showing that the PMMA resonances at 1730 and 1445 cm^−1^ fade with *x* as the water thickness *d* increases while the 1644 cm^−1^ resonance of water increases, (**D**) peak heights (data points) determined from **c** for the most prominent lines of PMMA and water, black and blue, respectively, *vs* the water depth *d* (see Supplementary Fig. S3b). Theoretically predicted full curves in (**D**) are calculated using the multi-layer model of near-field interaction^59^ (“Methods”), assuming a 100 nm tip radius and 70 nm tapping amplitude, and plotted × 1.78 in order to match the experimental PMMA peak height at sphere centre.

Here, we show that well-defined polymer spheres suspended in water below SiN can be chemically recognised up to a sizable depth of submersion. Figure 4A and Supplementary Fig. S3 depict the simultaneously recorded infrared amplitude *s*_2_, mechanical phase *φ*_mech_ and topography *z* images of a 10-µm diameter PMMA sphere adhering to the SiN membrane in water. Both show a clear contrast that allows to localise the PMMA sphere outlined in Fig. 4A as a dashed circle. The topography (Supplementary Fig. S3b) shows that the membrane bulges up by few nm where the PMMA spheres are centred and sinks in further out by also few nm due to adhesion forces. A line scan of nano-FTIR spectra taken along a radius *x* (Fig. 4B,C) shows good agreement with published FTIR spectra of water and PMMA, verifying that the SiN-based nano-FTIR platform allows to chemically identify materials underneath the SiN membrane.

The effect of a water space between the membrane and a submerged object can be quantified by extracting the peak heights of the main resonances, in case of water and PMMA from the spectra in Fig. 4C, and plot them *vs* the thickness *d* of the water layer (Fig. 4D).

The depth *d* corresponding to the experimental dots in Fig. 4D is determined from *x* by first calculating a nominal depth *d*_*nom*_ under the assumption that the sphere touches at just one central point, then correcting the result by the simultaneously measured topography of the membrane which bulges up by a few nm and, in a similar fashion, sinks in further outwards by also a few nm (Supplementary Fig. S3). The experimental dots in Fig. 4D determine that the PMMA signal reaches half of its maximum value at *d*_*1/2*_ = 70 nm (dashed red vertical line). An important conclusion follows for the study of living cells, namely that SiN-membrane based s-SNOM is able to investigate deeply into the interior of cells.

The theoretical calculations assume a 100 nm tip radius in order to match the experimental value of *d*_*1/2*_ = 70 nm. Note that *d*_*1/2*_ scales roughly proportional to the tip radius (Supplementary Fig. S3d). Extrapolating, we expect that sharper tips with radii around 15 nm could bring *d*_*1/2*_ down to about 10 nm which may enable an interesting infrared-spectroscopic study of interface-promoted ordering in extremely thin water layers, recalling that rotational immobilisation of water near *d* = 1 nm was recently established by tip-confined microwave probing^26^.

Furthermore, we note a very slight red-shift of the 1730 cm^−1^ line of PMMA which becomes visible in Fig. 4C as the PMMA surface submerges deeper than *d* ≈ 20 nm into water, an effect established in a recent study of PMMA buried under PS^27^.

### Individual *E. coli* cells monitored in their development

For a first biological object, we demonstrate the observation of living *E. coli* bacteria cells. The organisms were taken from a culture maintained at 37 °C and incubated for 30 min in an open SiN cell at 37 °C, 5% CO_2_ content, and 95% humidity. Then the cell was sealed by a glass cover slide and brought into the lab at room temperature (22 °C) for immediate s-SNOM measurement.

**Figure 5:**
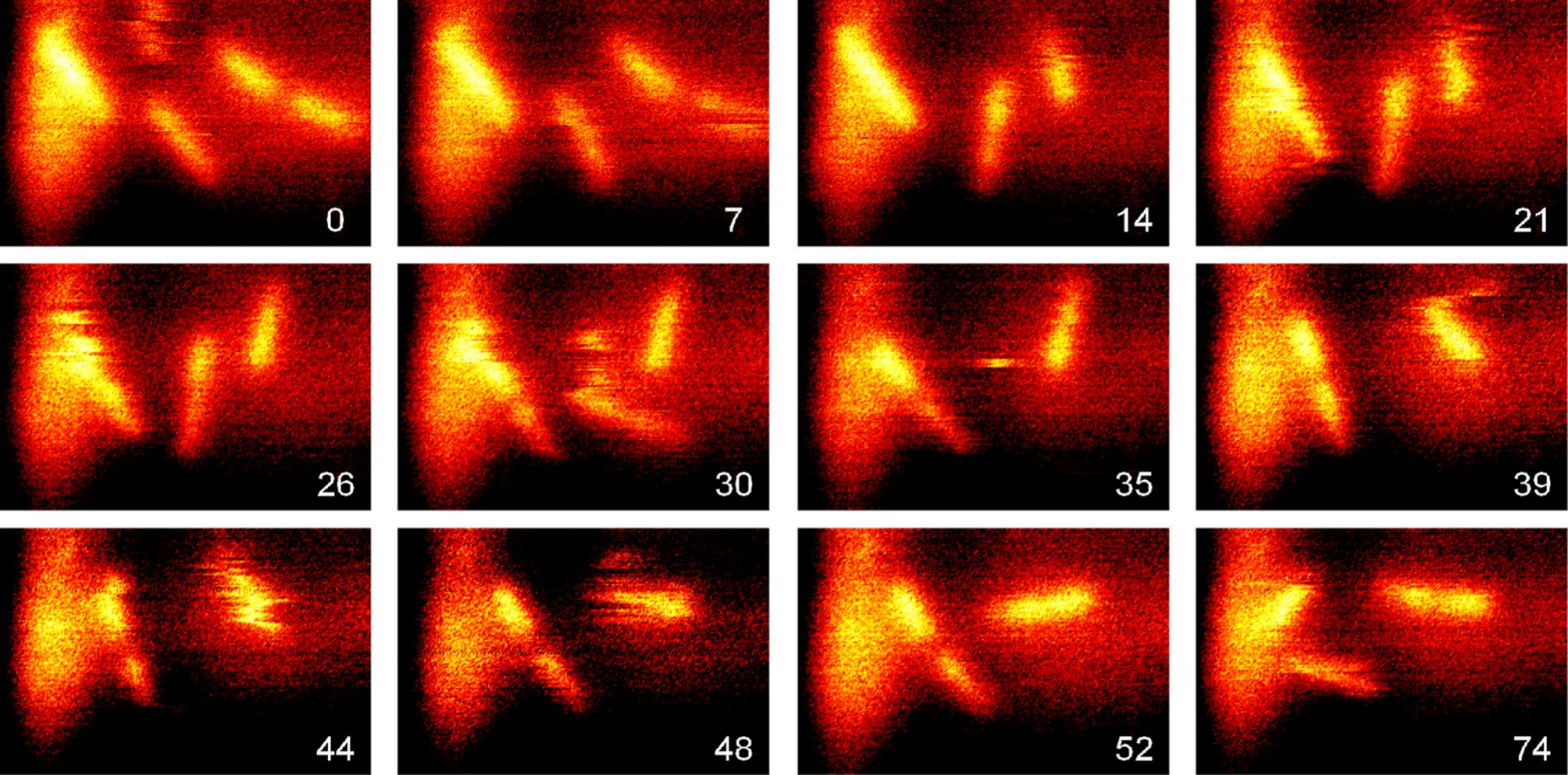
Adhesion-localised, but mobile living *E. coli* cells, sequential s-SNOM infrared amplitude *s*_*2*_ images as cells adhere, grow and move in the medium under a 15 nm SiN membrane (Norcada NBPX5002YZ-HR), at times (min) indicated, color scale as in Fig. 4A, image size 7.5 × 5 µm^2^.

The infrared images in Fig. 5 show that the cells are oriented mostly parallel to the membrane and appear brighter than the surrounding water (note the infrared amplitude is an average over a 600 cm^−1^ wide (FWHM) spectrum centred around 1400 cm^−1^, see Supplementary Fig. S1). They seem about 400 nm (FWHM) wide. Yet the cells' adhesion forces to the membrane are so weak that no mechanical contrasts are registered, weak enough to permit numerous evident relocations within 4 min between consecutive images, but on the other hand strong enough to keep images mostly unblurred during the typical acquisition time of 0.5–1 s for a given cell. The upper left cell, for example, grows in length and seemingly divides at circa 30 min into parts which separate much later. The upper right cell turns circa 90° between 35 and 52 min, but in between its image appears fuzzy, with stripes along the direction of the scan that repeats every 3 s, indicating this cell changes position irregularly on a time scale of several s. Lastly, a third cell seen in the middle of the image at 35 min, detected for a few consecutive scans only, begins to look fuzzy in the 30 min image after it had been in a well-defined shape for the preceding half hour. In the future such dynamics could be tracked with orders-of-magnitude higher frame rate, by using optical-parametric or quantum-cascade laser sources^5,28,29^.

### Observation of strongly adhering *E. coli* that exhibit modified infrared spectra

**Figure 6:**
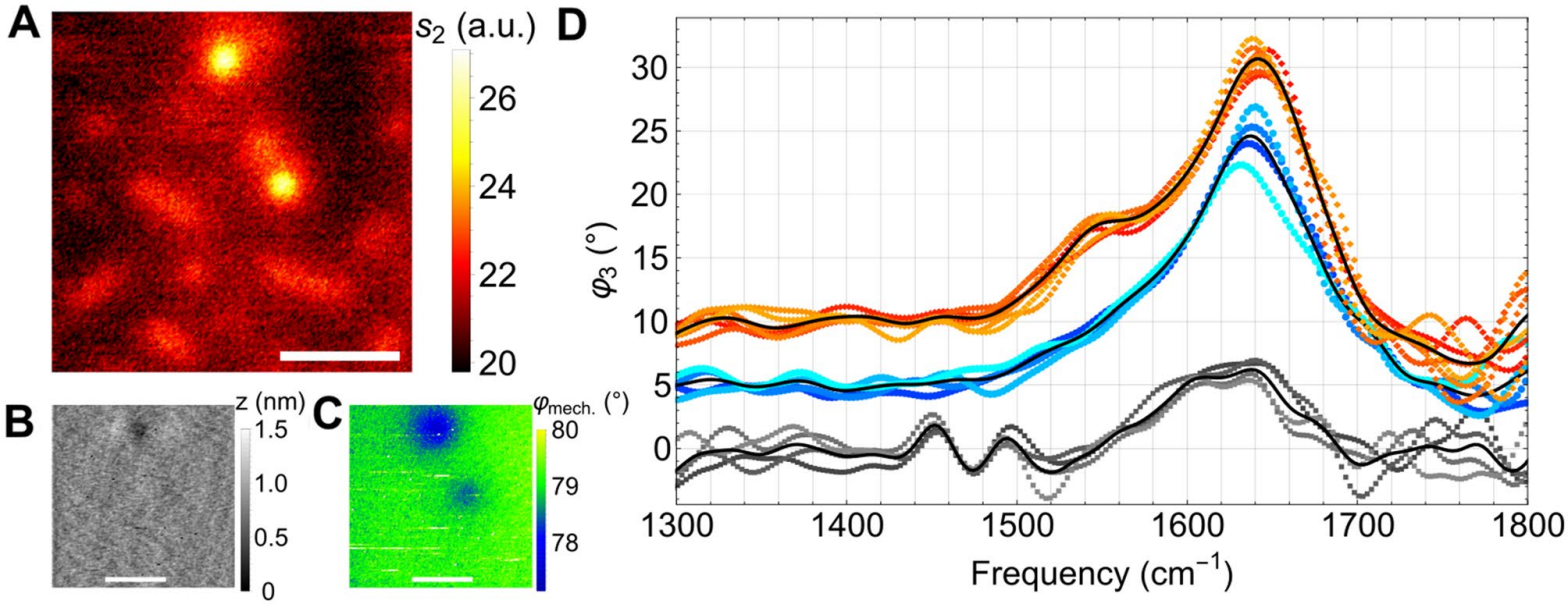
Living *E. coli* cells characterised by s-SNOM and nano-FTIR, (**A**) infrared amplitude *s*_*2*_ image of a preparation as in Fig. 5 exhibiting two bright, round cells which also appear in the simultaneously recorded mechanical images of (**B**) topography and of (**C**) mechanical phase *φ*_*mech*_, scale bars 2 µm. (**D**) Nano-FTIR phase *φ*_*3*_ spectra (data points) with averages (lines, offset 5° each for clarity), taken on bright round cells (black), lengthy cells (red), or between cells (blue) of (**A**), directly prove significantly different spectra, and thus chemical content, when probing a side *vs* a front location of a cell's envelope.

Surprisingly, *E. coli* images as in Fig. 6A can reveal cells which are distinctly brighter in the infrared and seemingly adhere head-on, with an apparent width of circa 500 nm. Only these show an effect on the mechanical images, namely a local topographic depression of about 0.5 nm (Fig. 6B), and a mechanical phase decrease of about 1° (Fig. 6C), both with an apparent width of also ca. 500 nm. This indicates that they adhere more tightly to the SiN membrane. The mechanical phase contrast in tapping mode AFM records the lag of the actual tip oscillation from its electrical drive, due to stiffness and adhesiveness of the surface^30,31^. Both properties are intrinsic to a material and therefore they could not be changed, in case of the SiN membrane, by the adhering cells on their back surface. Consequently we have discovered with Fig. 2C a different, novel contrast mechanism of AFM. With this method, one can take advantage of an extrinsic mechanical load on a thin membrane's back surface locally influencing the tapping phase in an unexplored fashion that suggests theoretical considerations beyond the scope of this work. Suffice it to say that mechanical contrasts constitute a valuable correlative information for s-SNOM images, providing insight into the adhesion forces between objects and membrane. It is known that *E. coli* may weakly adhere to abiotic surfaces by van der Waals forces in a non-specific manner, but also that, when sensing surfaces, peripheral cell components such as exposed proteins, pili or flagella can locally initiate complex mechanisms to stimulate the synthesis of adhesins, which generate a more permanent type of attachment^32^.

Infrared near-field spectra offer additional clues by determining local chemical compositions. A big surprise is that the head-on spectra (black in Fig. 6D) differ drastically from those side-on. The former show peaks at 1445 cm^−1^ assignable to lipids^33,34^, and at 1495 cm^−1^ to lipids or amino acids^35^, but largely seem to lack the proteins' amide I and II bands^35^ at 1640 and 1550 cm^−1^, respectively. In contrast, the latter two protein bands dominate the side-on spectra (red), but with an amid I/II ratio higher than generally reported in the literature^33,36^. We conclude that water must be present in the near-field interaction volume, considering that its bending-vibrational line (see blue in Fig. 6D, and also Supplementary Fig. S2) coincides with the amide I band. By continuing this assumption, the head-on spectra clearly indicate a smaller water content. A detailed analysis of the *E. coli* near-field spectra is beyond the scope of our present study, but we remark that our preparation method could be extended to perform controlled dehydration studies by in situ removing water, to arrive at local spectra which could be directly compared to state-of-the-art spectra of dry, embedded cells^5^. Further on, water's distortion of the protein amide I/amide II fingerprint could be totally avoided by exchanging H_2_O for D_2_O in the cell suspension.

### Correlative adhesion and infrared contrasts of a living cancer cell

**Figure 7:**
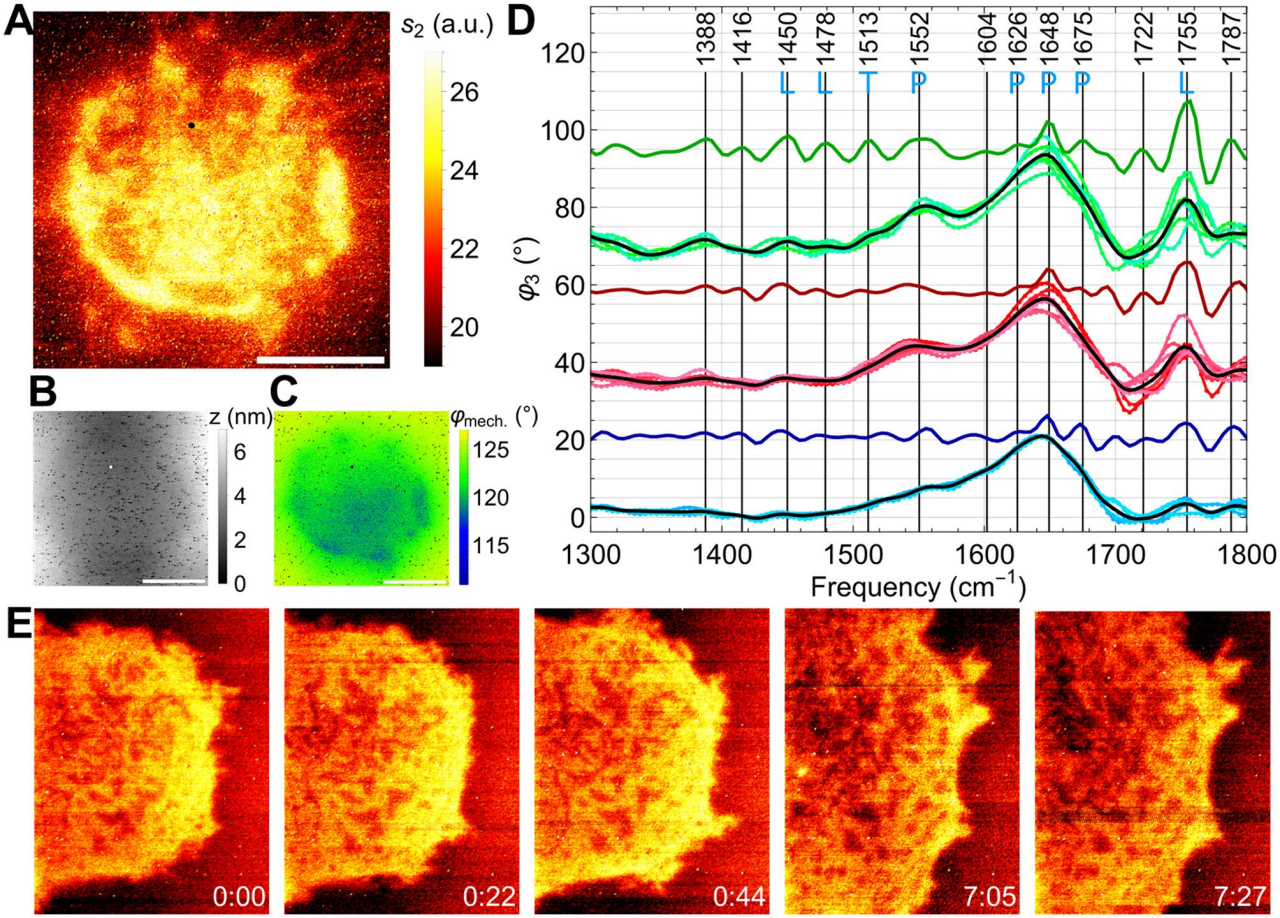
s-SNOM images and nano-FTIR spectra of living A549 cancer cells, (**A**) infrared amplitude *s*_*2*_ image taken 5 h after drop-casting the cell suspension on the 10-nm SiN membrane (250 × 250 µm^2^, Norcada NX5025Z), (**B**) simultaneously registered topography *z* and (**C**) AFM phase *φ*_*mech*_ images exhibiting patches interpreted as adhesion footprint, scale bars 5 µm; (**D**) nano-FTIR phase spectra *φ*_*3*_ at a few arbitrary positions within a bright peripheral spot (green data points, taken in 4 min each), or in between such spots (red), or outside the cell's footprint (blue); average spectra (black curves) and their calculated negative 2nd derivatives (coloured curves), with frequencies stated for the peaks in the green curve together with their assignments as P-protein, L-lipid, T-tyrosine, (**E**) infrared amplitude *s*_*2*_ images (11.6 × 15.1 µm^2^) of another A549 cell, corrected for sample drift, continuously acquired for 7:27 h in 22 min sequence starting 5 h after drop-casting, of which the first three and the last two are shown.

Nano-imaging of a human cell (Fig. 7) from the A549 lung carcinoma line proceeds likewise in similar, simple preparation steps of trypsin detachment from a culture flask at 37 °C, resuspension in nutrient Leibovitz's L-15 medium with 10% qualified FBS from Gibco at a concentration between 10^5^ and 10^6^ cells/ml (see “Methods”), drop-casting into an open SiN cell, and incubating for 1 h at 37 °C, 5% CO_2_ content, and 95% humidity. Then the cell was sealed by a glass cover slide and brought into the lab at room temperature (22 °C) for immediate s-SNOM measurement.

A suitably isolated cell selected in the NeaSNOM's overview microscope appears in nanoscopic images (Fig. 7A–C) not only with highly structured infrared contrast, but also with rich mechanical phase contrast and only a shallow topographical deformation of < 1 nm. Adhesion patches of cells similar to Fig. 7C are known from interference-contrast optical microscopy^37^, and of A549 cells by immunostaining^38^. Regardless of whether the observed cell's mechanical footprint in Fig. 7B,C is due to adhesion forces alone or influenced by more parameters, the fact that µm-sized patches and fine structures develop with time makes the correlative mechanical images a perfect addition to the infrared images for assessing the dynamics of a cell^39^. The mechanical images do not resolve the finest morphologic detail that is well visible in the infrared image, inside as well as outside the adhesion footprint. The missing mechanical contrast may be caused by a relatively weaker local adhesion of such fine structures. The outside fine structures seemingly correspond to well-known cell extensions.

### Assessing a single cancer cell's growth and chemical inhomogeneity

Evidently, the cell observed in Fig. 7E does not move, as can be judged from numerous tiny markers visible in all images of Fig. 7, which (apart from a single dust particle on top of the membrane in Fig. 7A–C) originate from random dimples in the membrane's upper surface that appear in infrared images as bright spots. They can serve as convenient criteria to align long sequences of imaging for thermal drifts of the sample stage, as is done here. The images in Fig. 7E clearly document that the cell grows in all directions, while numerous interior and peripheral morphological details undergo continuous changes. For example, some protrusions appear from one to the next image while others disappear. Again any interpretation would be beyond this paper's scope. However, these images already illustrate the potential of SiN-based nanoscopy to study cell locomotion^39^.

As to the spectroscopic dimension enabled by nano-FTIR nanoscopy (Fig. 7D), the infrared spectra taken outside the cell's footprint (blue dots) exhibit the expected dominating H_2_O vibrational resonance at 1644 cm^−1^, comparable to Figs. 3 and 6D. The spectra are highly reproducible, varying by no more than ± 3% in peak height, which we interpret as instrumental noise assuming that the water space is homogeneous. The spectra taken inside the cell's footprint (green and red dots) show significant contents of protein at 1550 cm^−1^ (amide II)^35^ and of lipids at 1755 cm^−1^, respectively^36^. As with the *E. coli* spectra (Fig. 6D), the presence of the amide II band calls for an amide I contribution at 1640 cm^−1^, however less than what is actually observed. Hence, we again conclude that H_2_O must also be present. A highly surprising observation is that the spectral peak heights, best judged from the water/amide I peak around 1644 cm^−1^, vary by ± 20% at neighbouring positions and thus document a high, position-dependent variation of the local chemical composition. Therefore, zoomed imaging paired with spectroscopic line scans, or better, 2-d hyperspectral images^27,40^ in a future study should be successful and rewarding in chemically recognising and characterising organelles and other sub-cellular entities. Since the few characteristic frequency positions of important molecules are well known, this kind of imaging could be accelerated from minutes to seconds by using step-tuned narrow-band lasers such as QCLs^5,29^.

Similarly as with common diffraction-limited FTIR studies of cells (at circa 3 µm spatial resolution)^5,41,42^, post-processing raw spectra by calculating the 2nd derivatives in respect to frequency (Fig. 7D) can distinguish and quantify α-helix (1648 cm^−1^) and β-pleated sheet (1626 and 1675 cm^−1^) secondary-structure abundances of the probed protein content. In principle, further analysis by Fourier self-deconvolution can disentangle overlapping resonances^43^. Clearly the nano-FTIR technique could identify different protein substructures in living organisms, and thus provide structural information together with chemical information. The 2nd derivative spectra also help assign the weaker peaks, such as the ones at 1450 and 1478 cm^−1^ to lipids, and that at 1513 cm^−1^ marked "T" to tyrosine^33^.

## Discussion

In summary, we have combined nanometric spatial resolution, chemical specificity, and liquid-sample operation to realise a general, non-perturbing, infrared-spectroscopic super-resolution microscopy technique.

Considering biology as the most interesting application area, we have demonstrated that unlabelled living cells can be imaged in their inherent infrared vibrational contrasts, at 150 nm resolution. We detect the movements of cells, track cells separating, and see inside cells. We obtain clear near-field spectra which agree with theory calculations and measure the relative abundances of protein, lipid and water. We observe that spectra and thus chemical content vary considerably within narrow neighbourhoods inside a cell's footprint. No sample preparation step such as fluorophore labelling, fixation, staining, or drying is involved in SiN-based s-SNOM. Of these, only labelling would make sense for observing living cells. Indeed, implementing a far-field fluorescence channel into an s-SNOM is in principle possible but has not yet been tried to our knowledge.

The key element of chemistry- as well as of biology-ready infrared s-SNOM is the biocompatible, wettable, and smooth SiN membrane, which, on its underside, arrests cells by weak adhesion forces in a probably gentle, stress-free manner, such that they can be quasi-fixed or quasi-free. The liquid space is perfectly isolated from the outside and separately controllable in regard to temperature, purity, sterility, medium composition, and pH value. Filled liquid cells are long-term stable and can be easily stored and transported. Loading requires a simple drop-casting of a particle suspension, although a microfluidic system^42^ would in the future provide easy medium exchange or sequential chemical treatment.

The strict separation of observer and object spaces and the SiN membrane's homogeneity and flatness enable an unmatched level of s-SNOM imaging quality and process standardisation. The absence of topography minimises image artefacts, spares tips from breaking, and enables high-speed scanning. The absence of disturbing variations encountered with uncovered samples—be it in elasticity, stickiness, contamination, or water adsorption—enables, on SiN, a continued operation at optimal, fixed AFM parameters (mechanical set point and feedback gain) and optimal, fixed infrared alignment. All can remain untouched with any sample exchange, thus rendering observed signals and spectra quantitatively repeatable, as already verified here. Note in passing that imaging through a flat membrane constitutes an interesting new mode of scanning microscopy, differing from most published AFM-based work where the tip follows the sample's topography ("constant distance"), by scanning a perfectly plane real surface (akin to the virtual "constant-height" plane of early SNOM work)^44^. In respect to depth profiling of molecular abundances, a further decisive advantage of the SiN membrane is that it provides a reproducible reference plane for standardisable, systematic variation of tapping parameters. Lastly, the observer space is tolerant regarding its environmental requirement including sterility, with the exception that low humidity is required to minimise condensation on the tip, and a low acoustic level is advantageous to minimise mechanical disturbances.

Water needs no longer be negatively connoted in nano-FTIR as is customary in FTIR studies of biological objects for its considerable and wavelength-dependent absorption loss and the problems of operating few-µm-path cuvettes and of coping with their interferences. In nano-FTIR water's vibrational resonance is well expressed (Supplementary Fig. S2), even though the interaction path length is of the order of 100 nm only, and thus much shorter than the far-field penetration depth of minimally 4 µm (on resonance). This comparison illustrates the counter-intuitive, general fact that near-field microscopy can and does cope with ultrasmall volumes of a material, producing identical amplitude and phase spectra even with arbitrarily small sample volumes, provided the tip radius can be chosen smaller than their spatial extent^6^.

NeaSNOM acquires all harmonic signal components up to 5th order and thus may enable a tomographic analysis^45,46^ that could in future extract veritable depth profiles of the dielectric function, and thus of layered materials or molecular abundances. Furthermore, the simple experimental design shown in Fig. 4 of progressively submerging an object could be extended to core–shell objects of different materials, such as coated vesicles, and lead to useful depth-related information. Our experimental findings prove (Fig. 4D) that a material's near-field spectrum taken through 70 nm of water retains 50% of its vibration-related modulation depth. This provides a centrally important message for the characterisation of living cells: SiN-membrane based nano-FTIR is able to investigate circa 100 nm deep into the interior of living cells, which are often surrounded by thick outer membranes that are far thicker than a basic 10-nm phospholipid bilayer.

With dielectric particles and living cells weakly adhering to the membrane's underside, we have discovered a new and useful contrast modality of AFM where adhering particles or cells induce a phase shift in the tapping oscillation, which we map as the cells' mechanical footprints.

Besides the presently demonstrated application with single cells, related biomedical interest could be found in tissue analysis, biomineralisation, and amyloid conformation and aggregation. Outside the life sciences, the combination of the SiN liquid cell and s-SNOM should permit unique studies in fields like catalysis, battery development, corrosion research, and wherever reactions or processes occur in aqueous environment at surfaces or interfaces. A reacting species could be held in place by either weak mechanical, optical or magnetic forces or by covalent tags, which allows for local chemical recognition to be tracked for long spans of time or down to a temporal resolution of circa 100 fs in case of repetitively triggered reactions^47^.

## Methods

### Cell culture

*E. coli* bacteria of the S-strain BZB1011^48^ were grown under standard conditions^49^. For the imaging experiments cells were taken from the log-phase of the culture corresponding to an optical density of OD600 ≈ 0.1.

A549 adeno carcinomic human alveolar epithelial cells were grown under standard conditions^50^. The cells were cultured at 37 °C, 5% CO_2_, and 95% humidity in Dulbecco's Modified Eagle Medium with GlutaMax by Gibco and supplemented 10% Fetal bovine serum (Gibco Qualified FBS). The cells were passaged every 2–3 days. For imaging, cells were detached from the bottom of the culture flask by incubating 5 min with a solution of 1 × 0.05% Trypsin–EDTA by Gibco. Next, the trypsin was deactivated by addition of L-15 Medium with 10% qualified FBS from Gibco, and the cells were separated from the deactivated trypsin solution by centrifugation. Afterwards the cells were resuspended at a concentration between 10^5^ and 10^6^ cells/ml in L-15 Medium with 10% qualified FBS. L-15 Medium was used in this step because it supports the growth of cells in an environment with low CO_2_ concentration, which is the case with our sealed liquid cell during the s-SNOM experiments. No antibiotics were added to the medium. Finally the cell suspension was drop-casted into an open SiN cell, and incubated for 1 h at 37 °C, 5% CO_2_ content, and 95% humidity. Then the cell was sealed by a glass cover slide and brought into the lab at room temperature (22 °C) for immediate s-SNOM measurement.

### Liquid cell design and usage

For filling in an aqueous suspension of cells or particles the SiN cell is turned upside down, and the suspension is simply drop-cast (Fig. 1C). Before, the device is irradiated for 20 min by UV-C to ensure its hydrophilicity. After filling it is advisable to wait circa 30 min for cells or other objects to sink by gravity and attach to the inner surface of the membrane. A standard microscopy cover slide seals the reservoir and thus completes the assembly of the SiN cell, which then is turned back so the membrane's dry surface comes uppermost, thus presenting no obstacle to the probing AFM-tip and any holding structures of the s-SNOM.

The temperature of SiN devices could conveniently be stabilised by a Peltier base plate. A complete filling of the SiN cell with liquid is not necessary because capillary forces keep water wetting the membrane independently of cell orientation. An air space within the cell should even be advantageous for oxygen supply, for example, of aerobic cells. This device can offer a well-defined and well-controllable microclimate of temperature, pressure and humidity that should be advantageous for studying moist biological systems or processes such as condensation on solid surfaces.

### s-SNOM imaging operation

The instrument (NeaSNOM from attocube.com) contains an AFM operating with standard cantilevered Pt-coated Si tips (attocube "nano-FTIR probes" having tip widths around 60 nm) in tapping mode, i.e., oscillating vertically at circa 70 nm amplitude and 300 kHz frequency (double arrow in Fig. 1a). The mechanical images comprise topography *z* and phase *φ*_*mech*_ of tip oscillation. The basic instrument is complemented by an infrared observation channel, consisting of a light source based on difference-frequency generation, (DFG, see next paragraph), a parabolic mirror (not shown) to focus the light beam on the AFM tip and to collimate back-scattered light, and a Michelson interferometer to determine both amplitude *s* and phase *φ* of the backscattered light into separate signal channels. The focusing parabola has an effective focal length of 15 mm, the uncoated parallel-plate ZnSe beam splitting mirror (BS) has a single-surface reflectivity of 28% for any mid-infrared wavelength up to circa 20 µm (polarised with E-field in the plane of Fig. 1, at 45° incidence), and the MCT detector (D) has a slightly rising responsivity for any mid-infrared wavelength up to circa14 µm. The electronics registers not only the direct detector signal, but in addition its modulated components at the first five harmonics of the tapping frequency all at once. Usually low-harmonic-demodulation signal components (*s*_*n*_ and *φ*_*n*_ with n = 2 or 3) are published because they have the "background" scattering well suppressed. They are usually normalised to reference data taken on a Si sample (see also Supplementary Fig. S1)^8^. In this work, we acquire overview infrared images by setting the movable reference mirror R at its "white light" distance from the beam splitter such that it equals the distance between beam splitter and tip, hence all spectral components of the source add constructively and the detector signal becomes the average amplitude weighted according to the spectral power profile. A typical integration time per pixel is 4 ms, to obtain a signal-to-noise ratio S/N ≈ 200 on Si, and S/N ≈ 40 on *E. coli* under SiN membrane, and with this acquire the infrared image quality as in Fig. 6A of 40.000 pixel in 3 min.

### Nano-FTIR spectroscopic operation

The interferometer enables s-SNOM operation at widely separate wavelengths, especially nano-FTIR (Fourier-transform infrared) spectroscopy. It attains continuous spectral coverage using a difference-frequency-generated coherent source (DFG)^7,51^ supplied together with the NeaSNOM, of up to an octave in the mid-infrared "fingerprint" region. This source emits a circa 1 mW infrared beam in form of 100 fs pulses at circa 80 MHz repetition frequency. Its spectrum (Supplementary Fig. S1) is a quasi-continuum of sharp lines at frequencies that are all integer multiples of the repetition frequency (harmonic frequency comb). The pulse nature allows to determine changes of near-field-scattering during ultrashort observation times between 100 fs and 10 ns or even longer, after laser triggering a repetitive material excitation^52^, in principle of interest for future studies in biology. Nano-FTIR spectra are obtained by moving mirror R periodically for a distance *x*, typically 300 µm, followed by online Fourier transformation, to achieve an instrumental spectral resolution of 1/2*x* = 17 cm^−1^ nominally. A typical scan time is 6 s, repeated typically 40× for averaging to obtain high S/N quality, short enough that usually no phase tilt from thermal drift in the interferometer needs to be corrected. Stronger infrared illumination^53^ could increase the S/N ratio so that spectra could be acquired much faster, as required for hyperspectral mapping^40^. Also, application of NeaSNOM's special signal processing routines such as synthetic holography^54^ would additionally help faster acquisitions. Note we usually plot the directly measured phase of scattered light which for polymers and biomolecules is proportional to absorbance^9,13^.

### Theory to predict nano-FTIR spectra of multi-layer samples

Complex-valued back-scattering (in phase and amplitude), as routinely acquired in the n = 0–5 demodulation orders by NeaSNOM and nano-FTIR, can be approximately described by the finite-dipole (FD) model of near-field interaction^55^. The probing tip in this analytic model is described by an elongated spheroid with two open parameters that can be determined either by comparing with experiments measuring absolute scattering (in distinction to the commonly used normalisation against a "reference" material such as high-resistivity Si)^23,56^, or by numerical simulation which considers the geometry of tip and shaft in detail^17,57^. In this paper we take frequent measurements on Si for normalising the measured data, and accordingly normalise also all calculated data to (calculated) Si data (see also Supplementary Fig. S3).

Sub-surface material recognition by s-SNOM and nano-FTIR has established that the effective depth of probing relates to the tapping amplitude, and decreases with demodulation order^58^. For calculating the optical near-field response of multilayer samples with arbitrary thicknesses, the FD model has been further developed by using optical transfer matrices for the electrostatics limit, considering point charges plus image charges induced in each layer^59,60^. This method is computationally efficient. All it requires for input is each layer's thickness and dielectric function. We use these referenced dielectric data, for water^61^, PMMA^62^, and SiN^25^.

### Nano-FTIR spectra interpretation

Diffraction-limited FTIR spectroscopy is an established technique in many different biomedical research settings such as monitoring the metabolic response of organisms^63^, studying the dynamics of isolated protein^64^, cancer metastasis^65^, cellular differentiation^66^ and antibiotic resistance^67^. These studies are built on resonances of the four major molecular classes in cells (proteins, lipids, nucleotides, and polysaccharides) and use principal components analyses of spectral data^40,68,69^. Proteins have two major resonances, the amide I at 1655 cm^−1^ and the amide II at 1545 cm^−1^^33,35,36^. The former is of special interest because its structure reveals the secondary structure of the investigated protein^35^. Studying changes in spectra of the amide I band^70^ can therefore give an understanding of the action of drugs or toxins on proteins^64^. Assessing a protein's amide I band is however extremely difficult in the presence of water which exhibits an overlapping resonance. Lipids can be spectroscopically identified by an intense line near 1740 cm^−1^ originating from a carbonyl vibration of the fatty acid ester motive^33,36,71,72^. Further lipid resonances at 1470 cm^−1^, 1450 cm^−1^ and 1400 cm^−1^ are ascribable to vibrations of the C-H bond in the hydrocarbon backbone and of free carboxylic acid in fatty acids^33,36,71,72^. Important spectral signatures of nucleotide structures are around 1220–1250 cm^−1^ belonging to the asymmetric phosphodiester stretching vibration, at 1085 cm^−1^ belonging to the respective symmetric phosphodiester stretching vibration, and at 968 cm^−1^ originating from the C–O phosphodiester residue^33,36^. Infrared resonances for sugar residues are difficult to assign to specific vibrations but are usually found in the range of 900–1200 cm^−1^, dominated by C–O–C, C–O, and ring vibrations^71^.

## Supplementary Information

Supplementary Information.

## Data Availability

Full Data are available from K.K., korbinian.kaltenecker@neaspec.com.

## References

[1] Lane N (2015). The unseen world: Reflections on Leeuwenhoek (1677) 'Concerning little animals'. Philos. Trans. R. Soc. Lond. B Biol. Sci..

[2] Sambongi Y (1999). Mechanical rotation of the c subunit oligomer in ATP synthase (F0F1): Direct observation. Science.

[3] Hell SW, Wichmann J (1994). Breaking the diffraction resolution limit by stimulated emission: Stimulated-emission-depletion fluorescence microscopy. Opt. Lett..

[4] Betzig E (2006). Imaging intracellular fluorescent proteins at nanometer resolution. Science.

[5] Kallenbach-Thieltges A (2020). Label-free, automated classification of microsatellite status in colorectal cancer by infrared imaging. Sci. Rep..

[6] Knoll B, Keilmann F (1999). Near-field probing of vibrational absorption for chemical microscopy. Nature.

[7] Amarie S, Ganz T, Keilmann F (2009). Mid-infrared near-field spectroscopy. Opt. Express.

[8] Keilmann, F. & Hillenbrand, R. In *Nano-Optics and Near-Field Optical Microscopy* (eds Zayats, A. & Richards, D.) (ArtechHouse, 2009).

[9] Huth F (2012). Nano-FTIR absorption spectroscopy of molecular fingerprints at 20 nm spatial resolution. Nano Lett..

[10] Chen X (2019). Modern scattering-type scanning near-field optical microscopy for advanced material research. Adv. Mater..

[11] Dominguez G (2014). Nanoscale infrared spectroscopy as a non-destructive probe of extraterrestrial samples. Nat. Commun..

[12] de Los Santos Pereira A (2020). Conformation in ultrathin polymer brush coatings resolved by infrared nanoscopy. Anal. Chem..

[13] Amenabar I (2013). Structural analysis and mapping of individual protein complexes by infrared nanospectroscopy. Nat. Commun..

[14] Woerman AL (2015). Propagation of prions causing synucleinopathies in cultured cells. Proc. Natl. Acad. Sci. USA.

[15] Khatib O (2015). Graphene-based platform for infrared near-field nanospectroscopy of water and biological materials in an aqueous environment. ACS Nano.

[16] Yuk JM (2012). High-resolution EM of colloidal nanocrystal growth using graphene liquid cells. Science.

[17] de Jonge N, Ross FM (2011). Electron microscopy of specimens in liquid. Nat. Nanotechnol..

[18] Durmaz, Y. C., Goetz, A. & Keilmann, F. In *International Conference IRMMW-THz 2019* (eds Sirtori, C. & Tignon, J.) (2019).

[19] Lu YH (2019). Infrared nanospectroscopy at the graphene-electrolyte interface. Nano Lett..

[20] Lu YH (2020). Ultrathin free-standing oxide membranes for electron and photon spectroscopy studies of solid-gas and solid-liquid interfaces. Nano Lett..

[21] Meireles LM (2019). Synchrotron infrared nanospectroscopy on a graphene chip. Lab Chip.

[22] Tselev A, Velmurugan J, Ievlev AV, Kalinin SV, Kolmakov A (2016). Seeing through walls at the nanoscale: Microwave microscopy of enclosed objects and processes in liquids. ACS Nano.

[23] McArdle P, Lahneman DJ, Biswas A, Keilmann F, Qazilbash MM (2020). Near-field infrared nanospectroscopy of surface phonon-polariton resonances. Phys. Rev. Res..

[24] Bunch JS (2008). Impermeable atomic membranes from graphene sheets. Nano Lett..

[25] Cataldo G (2012). Infrared dielectric properties of low-stress silicon nitride. Opt. Lett..

[26] Fumagalli L (2018). Anomalously low dielectric constant of confined water. Science.

[27] Mester L, Govyadinov AA, Chen S, Goikoetxea M, Hillenbrand R (2020). Subsurface chemical nanoidentification by nano-FTIR spectroscopy. Nat. Commun..

[28] Mörz F (2017). Nearly diffraction limited FTIR mapping using an ultrastable broadband femtosecond laser tunable from 133 to 8 µm. Opt. Express.

[29] Pfitzner E, Heberle J (2020). Infrared scattering-type scanning near-field optical microscopy of biomembranes in water. J. Phys. Chem. Lett..

[30] Lavini F (2020). Atomic force microscopy phase imaging of epitaxial graphene films. J. Phys. Mater..

[31] Raghavan D, Gu X, Nguyen T, VanLandingham M, Karim A (2000). Mapping polymer heterogeneity using atomic force microscopy phase imaging and nanoscale indentation. Macromolecules.

[32] Berne C, Ellison CK, Ducret A, Brun YV (2018). Bacterial adhesion at the single-cell level. Nat. Rev. Microbiol..

[33] Lasch, P. & Naumann, D. *Encyclopedia of Analytical Chemistry* 1–32 (2015).

[34] Wood BR (2016). The importance of hydration and DNA conformation in interpreting infrared spectra of cells and tissues. Chem. Soc. Rev..

[35] Barth A (2007). Infrared spectroscopy of proteins. Biochim. Biophys. Acta.

[36] Stuart, B. H. *Encyclopedia of Analytical Chemistry* (2012).

[37] Limozin L, Sengupta K (2009). Quantitative reflection interference contrast microscopy (RICM) in soft matter and cell adhesion. ChemPhysChem.

[38] Kogan TV, Jadoun J, Mittelman L, Hirschberg K, Osherov N (2004). Involvement of secreted *Aspergillus fumigatus* proteases in disruption of the actin fiber cytoskeleton and loss of focal adhesion sites in infected A549 lung pneumocytes. J. Infect. Dis..

[39] Schreiber C, Amiri B, Heyn JCJ, Radler JO, Falcke M (2021). On the adhesion-velocity relation and length adaptation of motile cells on stepped fibronectin lanes. Proc. Natl. Acad. Sci. USA.

[40] Amenabar I (2017). Hyperspectral infrared nanoimaging of organic samples based on Fourier transform infrared nanospectroscopy. Nat. Commun..

[41] Kretlow, A., Lasch, J. K., Beekes, P. M., Miller, L. & Naumann, D. In *Biomedical Applications of Synchrotron Infrared Microspectroscopy* (ed Moss, D.) (2011).

[42] Vaccari L, Birarda G, Businaro L, Pacor S, Grenci G (2012). Infrared microspectroscopy of live cells in microfluidic devices (MD-IRMS): Toward a powerful label-free cell-based assay. Anal. Chem..

[43] Baker MJ (2014). Using Fourier transform IR spectroscopy to analyze biological materials. Nat. Protoc..

[44] Novotny, L. & Hecht, B. *Principles of Nano-Optics*. (2007).

[45] Taubner T, Korobkin D, Urzhumov Y, Shvets G, Hillenbrand R (2006). Near-field microscopy through a SiC superlens. Science.

[46] Sun J, Schotland JC, Hillenbrand R, Carney PS (2009). Nanoscale optical tomography using volume-scanning near-field microscopy. Appl. Phys. Lett..

[47] Wagner M (2014). Ultrafast dynamics of surface plasmons in InAs by time-resolved infrared nanospectroscopy. Nano Lett..

[48] Kerr B, Riley MA, Feldman MW, Bohannan BJ (2002). Local dispersal promotes biodiversity in a real-life game of rock-paper-scissors. Nature.

[49] Elbing KL, Brent R (2019). Recipes and tools for culture of *Escherichia coli*. Curr. Protoc. Mol. Biol..

[50] Cooper JR (2016). Long term culture of the A549 cancer cell line promotes multilamellar body formation and differentiation towards an alveolar type II pneumocyte phenotype. PLoS ONE.

[51] Keilmann F, Amarie S (2012). Mid-infrared frequency comb spanning an octave based on an Er fiber laser and difference-frequency generation. J. Infrared Millimeter Terahertz Waves.

[52] Wagner M (2014). Ultrafast and nanoscale plasmonic phenomena in exfoliated graphene revealed by infrared pump-probe nanoscopy. Nano Lett..

[53] Ru Q (2021). Two-octave-wide (3–12 µm) subharmonic produced in a minimally dispersive optical parametric oscillator cavity. Opt. Lett..

[54] Schnell M, Carney PS, Hillenbrand R (2014). Synthetic optical holography for rapid nanoimaging. Nat. Commun..

[55] Cvitkovic A, Ocelic N, Hillenbrand R (2007). Analytical model for quantitative prediction of material contrasts in scattering-type near-field optical microscopy. Opt. Express.

[56] Amarie S, Keilmann F (2011). Broadband-infrared assessment of phonon resonance in scattering-type near-field microscopy. Phys. Rev. B.

[57] Brehm M, Schliesser A, Cajko F, Tsukerman I, Keilmann F (2008). Antenna-mediated back-scattering efficiency in infrared near-field microscopy. Opt. Express.

[58] Taubner T, Keilmann F, Hillenbrand R (2005). Nanoscale-resolved subsurface imaging by scattering-type near-field optical microscopy. Opt. Express.

[59] Hauer B, Engelhardt AP, Taubner T (2012). Quasi-analytical model for scattering infrared near-field microscopy on layered systems. Opt. Express.

[60] Hauer, B. *Nano-optical mapping of permittivity contrasts and electronic properties at the surface and beneath doctoral thesis* (RWTH Aachen, 2015).

[61] Max JJ, Chapados C (2009). Isotope effects in liquid water by infrared spectroscopy. III. H_2_O and D_2_O spectra from 6000 to 0 cm^−^^1^. J. Chem. Phys..

[62] Hinrichs, K. *Infrared spectroscopic ellipsometry of PMMA (1100–1900 cm*^*−1*^*, Lucite, ISO 9002)* (ISAS, 2021).

[63] Holman HY (2009). Real-time molecular monitoring of chemical environment in obligate anaerobes during oxygen adaptive response. Proc. Natl. Acad. Sci. USA.

[64] Kruger A, Burkle A, Hauser K, Mangerich A (2020). Real-time monitoring of PARP1-dependent PARylation by ATR-FTIR spectroscopy. Nat. Commun..

[65] Bird B (2009). Detection of breast micro-metastases in axillary lymph nodes by infrared micro-spectral imaging. Analyst.

[66] Chen L (2012). Synchrotron infrared measurements of protein phosphorylation in living single PC12 cells during neuronal differentiation. Anal. Chem..

[67] Holman HY (2009). Real-time chemical imaging of bacterial activity in biofilms using open-channel microfluidics and synchrotron FTIR spectromicroscopy. Anal. Chem..

[68] Bird B (2012). Infrared spectral histopathology (SHP): A novel diagnostic tool for the accurate classification of lung cancer. Lab Investig..

[69] Chan KLA, Fale PLV, Atharawi A, Wehbe K, Cinque G (2018). Subcellular mapping of living cells via synchrotron microFTIR and ZnS hemispheres. Anal. Bioanal. Chem..

[70] Semenyshyn R (2019). In vitro monitoring conformational changes of polypeptide monolayers using infrared plasmonic nanoantennas. Nano Lett..

[71] Jackson M, Haris PI, Chapman D (1989). Fourier transform infrared spectroscopic studies of lipids, polypeptides and proteins. J. Mol. Struct..

[72] Mantsch HH, Chapman D (1995). Infrared Spectroscopy of Biomolecules.

