# Supplementary material for: Infrared-spectroscopic, dynamic near-field microscopy of living cells and nanoparticles in water
Source: Sci Rep. 2021 Nov 8;11:21860. doi: 10.1038/s41598-021-01425-w (PMC8576021; doi:10.1038/s41598-021-01425-w)
Supplement: Supplementary file 1 — Supplementary Information. [file 41598_2021_1425_MOESM1_ESM.pdf]

Infrared-spectroscopic, dynamic near-field microscopy of living cells and nanoparticles in water

Korbinian J. Kaltenecker[1,2], Thorsten Gölz[1], Enrico Bau[1], and Fritz Keilmann[1]*

[1]*Fakultät für Physik, Nano Institute Munich & Center for NanoScience (CeNS), Ludwig-Maximilians-Universität, Königinstr. 10, 80539 München, Germany*

[2]*present address: attocube Systems AG, Eglfinger Weg 2, 85540 Haar, Germany,*

F.K. email: fritz.keilmann@lmu.de

F.K. orcid.org/0000-0001-8334-2310

K.K. orcid.org/0000-0002-9831-6075

Supplementary Information

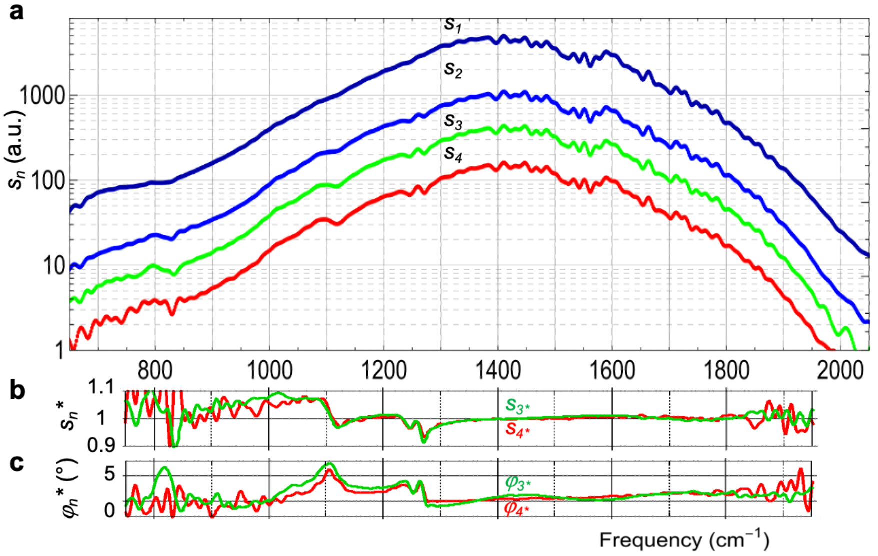

Supplementary Fig. S1. DFG source spectral range and normalisation, **a** raw nano-FTIR amplitude spectra s_n of demodulation orders n = 1-4 simultaneously measured on Si (commonly used as "reference" for normalising sample spectra, also in this work), acquisition time 10 min; their bell-shaped profile is due to the 1 mW coherent input beam from difference-frequency generation powered by pulsed Er fibre lasers; all spectra exhibit identical dips at >1300 cm^{-1} from atmospheric absorption along the circa 1 m path from the DFG source via the s-SNOM tip to the detector (their width of circa 13 cm^{-1} (FWHM) directly measures the spectral resolution of the NeaSNOM's interferometer).
In contrast, the spectra also exhibit features which are not constant but increase with n, at 820, 1100 and 1250 cm^{-1}. Therefore, they must originate from the near-field interaction. The systematic increase of spectral signatures with n has seemingly not been recognised in the nano-FTIR literature, but this effect is visible in published nano-FTIR spectra of SiO_2 (Figs. 4 of refs.[1,2]), for example, where phonon resonance signatures significantly increase from n=2 to n=3.
b Exploiting this effect for extracting the pure near-field resonances from a single, raw sample measurement—without referring to a reference spectrum—is proposed in the following way: *self-normalising* involves simply calculating self-normalised amplitude spectra $s_n^* = s_n/s_{n-1}$ and self-normalised phase spectra $\varphi_n^* = \varphi_n - \varphi_{n-1}$. This is exemplified in **b,c** for the Si spectra shown in **a.** Clearly, this simple normalisation procedure suppresses all atmospheric dips, circa 50-fold, and highlights three near-field resonances which turn out remarkably reproducible, even quantitative, for n=4 and 3, all being assignable to PDMS (see Fig. 5c in ref.[3]) which is a known contamination of commercial AFM tips and thus could, in principle, be avoided by cleaning tips. Note that such a normalisation has very recently been reported and shown by two groups to suppress in s-SNOM artefacts coming from independent scatterers that are in close proximity to the s-SNOM tip,[4] and /L. Mester&R. Hillenbrand, "High-fidelity infrared nanoimaging and nanospectroscopy by in-pixel signal normalization", under review/.

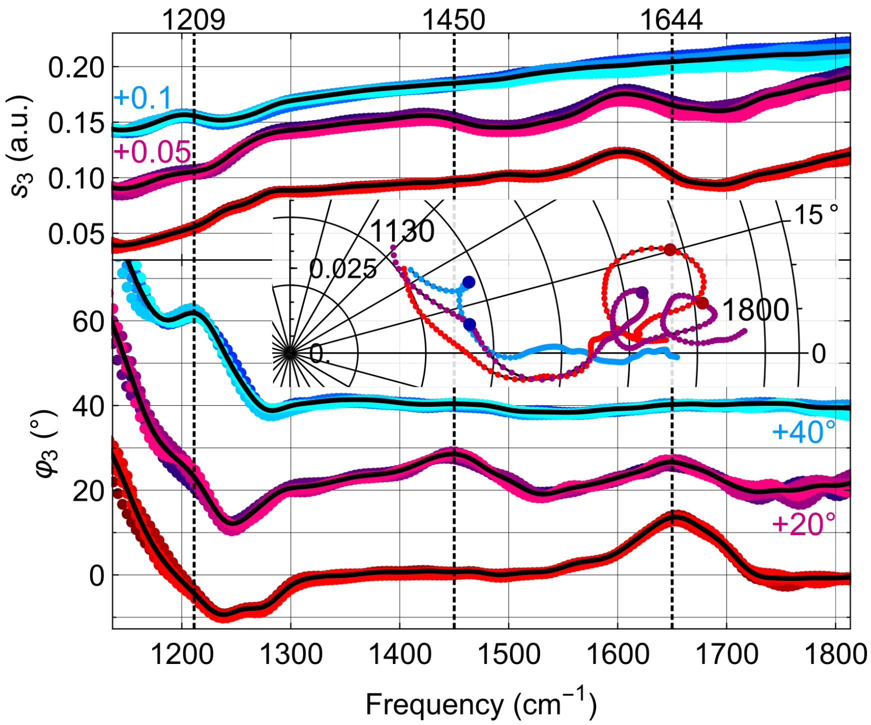

Supplementary Fig. S2. Nano-FTIR probing of water. Phase φ_3 spectra and amplitude s_3 spectra of H_2O (red), D_2O (blue) and 1:1 mixed (purple), covered by a 15 nm SiN membrane (Norcada NBPX5002YZ-HR), offset as indicated. Note that the equilibrated 1:1 mixture of H_2O and D_2O exhibits a 50% concentration of HDO, for statistical reasons. The resonances of H_2O, HDO and D_2O are depicted as black vertical lines at 1644, 1450 and 1209 cm^{-1}.[5]

These measurements determine for the first time the complex-valued nano-FTIR spectrum of water, for three different water isotopologues. They prove that the bending resonances approach circular traces in a polar plot (inset) where the resonance positions are marked by large dots. The traces appear strongly distorted by the SiN phonon in the low-frequency section around the D_2O resonance. Since the response of isotopically pure H_2O reduces to ca. 1/4 in the 1:1 mixture with D_2O, as predicted, this provides experimental evidence that relative molecular abundances can be quantitatively assessed by SiN-based nano-FTIR.

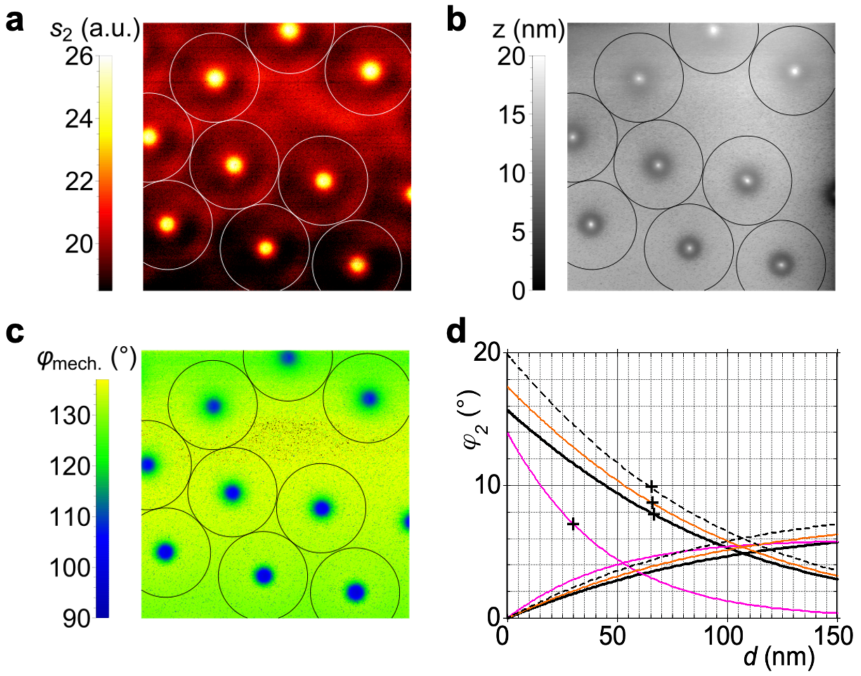

Supplementary Fig. S3. Calibration and prediction of depth sensitivity by s-SNOM imaging and nano-FTIR of water-suspended 10 µm diameter PMMA spheres (PMMA-F-10.0 from micro-particles.de), outlined as dashed circles, adhering below a 10 nm SiN membrane (Norcada NX5025Z), **a** infrared amplitude s_2 image, **b** simultaneously registered topography z and **c** AFM mechanical phase φ_{mech} images showing how adhesion forces modify the membranes' topography to bulge up by a few nm and to sink down further out by also a few nm;

d theoretically predicted peak heights φ_2 of PMMA (at 1730 cm^{-1}, decreasing curves) and of water (at 1644 cm^{-1}, increasing curves) for a water-suspended, 10 µm diameter PMMA sphere adhering below a 10 nm SiN membrane *vs* water thickness d (see Fig. 4b). The + symbols mark the point where each PMMA curve reaches half its maximum value, and thus allows to determine a characteristic depth $d_{1/2}$. The black full curve corresponds to the parameters (similar as used in Fig. 4) of a tip radius r = 100 nm and a tapping amplitude a = 100 nm. The red curve shows the result of decreasing the tip radius to r = 50 nm; clearly $d_{1/2}$ decreases approximately linear with r to about 45%. Increasingly sharp tips should therefore be used for near-surface investigations, while the opposite is true for probing possibly deep into a material. Variation of the other parameters entering the calculation have a much smaller effect on the characteristic depth $d_{1/2}$: the orange curve refers to a reduced tapping amplitude of a = 50 nm, while the black dashed curve refers to an increased demodulation order n = 3 (and thus to φ_3).

References

1 Amarie, S. & Keilmann, F. Broadband-infrared assessment of phonon resonance in scattering-type near-field microscopy. *Physical Review B* **83**, doi:10.1103/PhysRevB.83.045404 (2011).

2 McArdle, P., Lahneman, D. J., Biswas, A., Keilmann, F. & Qazilbash, M. M. Near-field infrared nanospectroscopy of surface phonon-polariton resonances. *Physical Review Research* **2**, doi:10.1103/PhysRevResearch.2.023272 (2020).

3 Huth, F. *et al.* Nano-FTIR Absorption Spectroscopy of Molecular Fingerprints at 20 nm Spatial Resolution. *Nano Lett.* **12**, 3973–3978 (2012).

4 McLeod, A. S. *et al.* Nano-imaging of strain-tuned stripe textures in a Mott crystal. *npj Quantum Materials* **6**, doi:10.1038/s41535-021-00339-0 (2021).

5 Max, J. J. & Chapados, C. Isotope effects in liquid water by infrared spectroscopy. III. H2O and D2O spectra from 6000 to 0 cm(-1). *J Chem Phys* **131**, 184505, doi:10.1063/1.3258646 (2009).
